# Clinical application of bone turnover markers in treating osteoporotic vertebral compression fractures and their role in predicting fracture progression

**DOI:** 10.1097/MD.0000000000029983

**Published:** 2022-08-12

**Authors:** Moon-Soo Han, Gwang-Jun Lee, Seul-Kee Lee, Jung-Kil Lee, Bong Ju Moon

**Affiliations:** a Department of Neurosurgery, Chonnam National University Medical School & Research Institute of Medical Sciences, Gwangju, Korea.

**Keywords:** bone turnover marker, osteocalcin, osteoporotic vertebral compression fracture, parathyroid hormone, teriparatide

## Abstract

This study aimed to investigate whether changes in the bone turnover markers (BTMs) during teriparatide therapy for osteoporotic vertebral compression fractures could reflect therapeutic effects by analyzing the relationship between clinical and radiological features and BTMs.

A total of 33 patients with 51 osteoporotic vertebral compression fracture segments were included. Plain radiographs and BTM levels were evaluated at the pretreatment and at 3 months after teriparatide treatment. Based on serial vertebral compression ratio analysis, the progression of fracture was defined as a vertebral compression ratio decrease of ≥10%, relative to the pretreatment values.

All segments were divided into 2 groups: the “maintain” group with 32 (62.7%) segments and the “progression” group with 19 (37.3%) segments. After the teriparatide treatment, serum osteocalcin and serum C-terminal telopeptide of type I collagen levels (*P* = .028 and .008, respectively), and change amounts of them were significantly larger, increasing (*P* = .001) in the progression group. The vitamin D (25OH-D) levels were significantly lower (*P* = .038) in the progression group; however, the relative changes in the 25OH-D levels between the 2 groups, before and after the treatment, were not significantly different (*P* = .077). The parathyroid hormone (PTH) levels were reduced by the teriparatide treatment in both groups, while the decrease in PTH concentration after the treatment was significantly more pronounced in the progression group (*P* = .006). Significant increase in the osteocalcin and serum C-terminal telopeptide of type I collagen levels and a simultaneous decrease in the PTH levels during the teriparatide treatment suggest that clinicians should assume the progression of fracture.

## 1. Introduction

Osteoporotic vertebral compression fractures (OVCFs) without neurological deficits are inherently stable fractures that involve only the anterior column of the vertebral body.^[1]^ Despite recent progress, a controversy exists regarding the best way to manage OVCFs. Teriparatide, a recombinant human parathyroid hormone (PTH), directly stimulates bone formation and improves bone strength and quality.^[2–5]^ Therefore, conservative treatment with teriparatide is considered the first-line treatment option for OVCFs without neurological deficits.^[4]^ However, the progression of compression fracture is not uncommon even after the conservative treatment of OVCFs and is difficult to predict or prevent.

Numerous bone turnover markers (BTMs) are the indicators of bone cell activity; their discovery has contributed to a marked improvement in the management of osteoporosis. BTMs are generally divided into 3 categories: bone formation markers (BFMs), bone resorption markers (BRMs), and osteoclast regulatory proteins.^[6]^ Several studies have suggested that BTMs, including BFMs and BRMs, could be used to evaluate bone healing and predict the progression of fracture.^[6,7]^ However, the use of BTM for prediction of progression of fracture is controversial, as there is a large variability between individuals due to difference in age and physiological maturity and the multiple methodologies used for analysis.

This study aimed to investigate whether changes in the BTMs during short-term teriparatide therapy for OVCF could reflect the therapeutic effects by analyzing the relationship between clinical and radiological features and BTMs. The potential risk factors for fracture progression using the pretreatment BTM levels were also evaluated.

## 2. Materials and Methods

### 2.1. Patient population and study design

This study was performed according to the requirements associated with patient anonymity and was approved by the Institutional Review Board of the Chonnam National University Medical School Research Institute, the Republic of Korea (CNUH-2019-366).

The patients who had undergone teriparatide treatment at least 3 months prior were included in this study. The treatment was performed using FORSTEO solution (Lilly, Indianapolis, IN) as a subcutaneous injection, in a prefilled pen, once daily for 3 months. Patients were required to wear thoracic-lumbo-sacral orthosis brace for 8 weeks. At the same time, daily oral supplementation with calcium carbonate (1250 mg) and cholecalciferol (1000 IU) in a tablet form was provided. To reduce the large variability between individuals at risk of fracture, patients were excluded based on fracture risk assessment tool (FRAX), male, premenopause female, a history of additional trauma of the fractured vertebra, administration of special medication regimens within the treatment period, such as corticosteroids, current smoker, alcohol use, patients unable to ambulate independently, incomplete records or imaging data, and inability to provide consent to participate in the study. Additionally, patients with other pathological fractures, such as chronic steroid administration, infective disease, metastasis, or myeloma, were excluded.

In total, 33 patients with 51 OVCF segments were included in this study. Patient’s clinical data at the time of treatment, such as data regarding age, height, weight, body mass index, presenting symptoms, medical history, medication use, bone mineral density (BMD), and radiological findings, were collected. Laboratory findings, including the details regarding the levels of serum telopeptide of type I collagen (CTX), serum osteocalcin (OC), calcium, phosphate, magnesium (Mg), PTH, alkaline phosphatase, and vitamin D (25OH-D), were also collected. Overnight fasting blood samples were drawn for laboratory assessment.

### 2.2. Radiological and clinical evaluation

**Figure 1.**
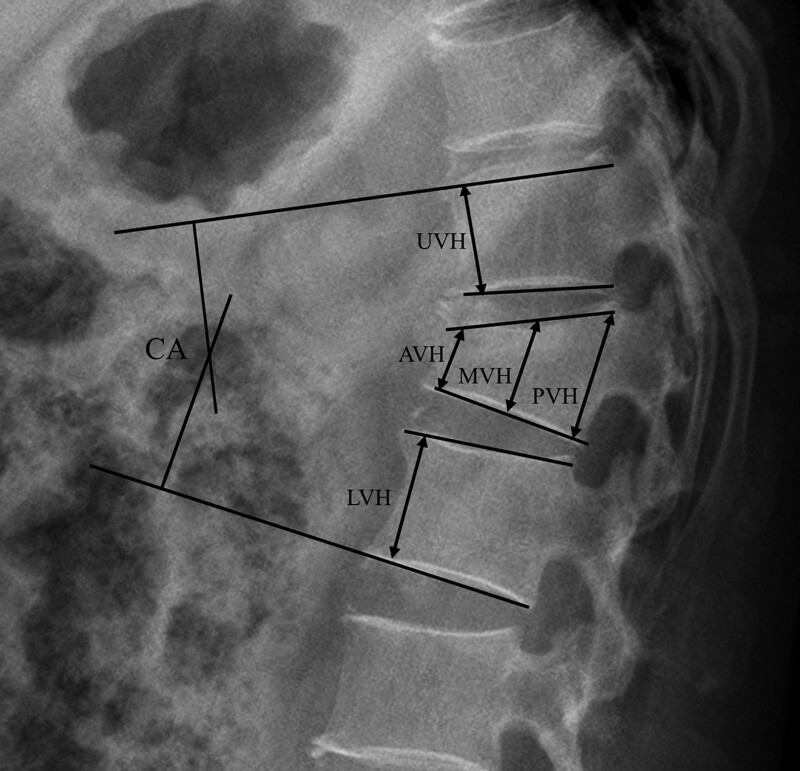
Parameters measured on a lateral radiograph. The vertebral compression ratio was calculated by the following formula: (1–[2 × AVH/UVH + LVH]) × 100. AVH = anterior vertebral height, CA = Cobb angle, LVH = lower vertebral height, MVH = middle vertebral height, PVH = posterior vertebral height, UVH = upper vertebral height.

Plain radiographs and BTMs were evaluated at the treatment stage and 3 months after conservative treatment with teriparatide. Local kyphosis was measured using the Cobb angle (CA), the angle between the superior endplate of the upper adjacent vertebra and the inferior endplate of the lower adjacent vertebra at the fractured vertebral body. Kyphosis was noted as a positive value, and lordosis as a negative value. The vertebral body height was measured at 3 points: anterior, middle, and posterior, between the upper and the lower end plates of the vertebral body. The vertebral compression ratio (VCR) was calculated as the ratio of vertical height of the most compressed portion of the injured vertebral body to vertical height of the same portion of the adjacent intact vertebral body. The following formula was used: VCR = (1 − [2 × fractured body height/ normal upper body height + normal lower body height]) × 100 (Fig. 1). VCR was measured at initial enrollment and 3 months after the compression fracture. All radiographs were evaluated by 2 independent surgeons. Based on serial VCR analysis, fracture progression was defined as a VCR decrease of ≥10% relative to the VCR at pretreatment at any of the 3 points measured.^[8,9]^ Based on this definition, we investigated the correlation between the BTM values and fracture progression.

To evaluate back pain, a visual analog scale (VAS) was used prior to treatment and at the last follow-up.

### 2.3. Statistical analysis

The outcome analysis was performed by comparing the data of the “progression” and “maintain” groups. All statistical analyses were performed using SPSS version 25.0 software for Windows (SPSS, Chicago, IL). Depending on the normality of the data, an independent sample *t* test was used to compare the parameters between the 2 groups, while a paired *t* test was performed to analyze the differences before and after the treatment. Categorical data, such as medical history and prior treatment for osteoporosis, were analyzed by a chi-square test. Logistic regression analysis was used to determine the correlation between the relevant factors and progression of compression fracture. *P* values of <.05 were considered statistically significant.

## 3. Results

### 3.1. Patient population

A total of 33 patients with 51 OVCF segments were included in this study. The mean follow-up period was 149.2 days (range: 121.1–193.4 days). The mean age of patients undergoing teriparatide treatment was 70.6 years (range: 59.3–81.38 years). The patients were divided into 2 groups based on their fracture status: a the “maintain” group with 21 patients and 32 (62.7%) segments, and the “progression” group with 12 patients and 19 (37.3%) segments. The clinical characteristics of the enrolled patients are summarized in Table 1; no significant differences in demographic characteristics were observed between these 2 groups.

**Table 1:** Summary of the baseline clinical characteristics of the patients.

Characteristics	Total (%)	Maintain group	Progression group	*P* value
No. of patients	33	21 (63.6%)	12 (36.4%)	
No. of segments	51	32 (62.7%)	19 (37.3%)	
Age (yr), mean (range)	70.6 (59.3–81.8)	68.6 (59.3–76.9)	72.5 (61.2–81.8)	.135
BMI (kg/m^2^)	23.9 ± 2.24	24.2 ± 3.24	23.6 ± 2.33	.527
BMD (g/cm^2^)	–2.99 ± 1.10	–3.03 ± 1.39	–2.94 ± 0.91	.807
Fracture level				.389
Thoracic	18 (35.7%)	10 (30.3%)	8 (42.1%)	
Lumbar	34 (24.3%)	23 (69.7%)	11 (57.9%)	
Prior osteoporosis treatment	7 (20.5%)	3 (13.6%)	4 (33.3%)	.224
F/U period, mean (d, range)	149.2	157.8	142.5	
	(121.1–193.4)	(121.2–193.4)	(123.1–161.9)	

### 3.2. Radiological and clinical outcomes

The progression of vertebral fracture was prominent on the anterior (*P* = .017) and middle (*P* = .005) portions of the vertebral body. There were no significant differences in the pretreatment VCR measurements between the groups (anterior, middle, and posterior: *P* = .574, .364, and .719, respectively); however, during the follow-up, the VCR increased significantly in the progression group (anterior, middle, and posterior: *P* = .012, .001, and .038, respectively) compared to the maintain group. There was a slight overall progression of local kyphosis in both groups, but there were no significant changes in the segmental angle at both pretreatment and follow-up. VAS showed overall improvement from 6.35 ± 1.17 to 3.25 ± 1.52 in all patients (*P* = .001), confirming a strong pain-relieving effect of teriparatide treatment in both groups (Table 2).

**Table 2:** Summary of radiological and clinical evaluations.

	Total	Maintain group	Progression group	*P* value*
VCR of anterior (%)				
Pretreatment	20.82 ± 23.84	19.39 ± 23.95	23.30 ± 24.08	.574†
F/U	25.81 ± 26.70	19.94 ± 25.01	37.92 ± 25.79	.012†
ΔF/U–pretreatment	4.99 ± 11.86	0.55 ± 5.77	14.62 ± 19.67	.004†
*P* value*	.017‡			
VCR of middle (%)				
Pretreatment	20.23 ± 21.10	18.19 ± 22.09	23.77 ± 19.32	.364†
F/U	24.95 ± 22.61	19.16 ± 21.92	38.38 ± 17.18	.001†
ΔF/U–pretreatment	4.72 ± 8.51	0.97 ± 6.85	14.61 ± 11.81	.000†
*P* value*	.005‡			
VCR of posterior (%)				
Pretreatment	8.09 ± 1.73	7.65 ± 12.93	8.88 ± 9.57	.719†
F/U	8.28 ± 12.98	9.84 ± 12.56	13.18 ± 12.52	.038†
ΔF/U–pretreatment	0.19 ± 7.25	2.19 ± 6.98	4.90 ± 9.85	.008†
*P* value*	.879‡			
CA, fracture level (°)				
Pretreatment	5.65 ± 18.06	5.00 ± 17.30	6.98 ± 20.06	.724†
F/U	7.51 ± 18.54	6.12 ± 17.20	8.86 ± 21.22	.383†
ΔF/U–pretreatment	1.86 ± 4.48	1.12 ± 3.55	1.89 ± 10.71	.289†
*P* value*	.380‡			
VAS				
Pretreatment	6.35 ± 1.17	6.27 ± 1.26	6.47 ± 1.02	.556†
F/U	3.25 ± 1.52	3.03 ± 1.38	3.63 ± 1.71	.172†
ΔF/U–pretreatment	–3.1 ± 1.35	–3.21 ± 1.52	–2.68 ± 1.83	.267†
*P* value*	.0001‡			

### 3.3. BTMs and laboratory outcomes

**Figure 2.**
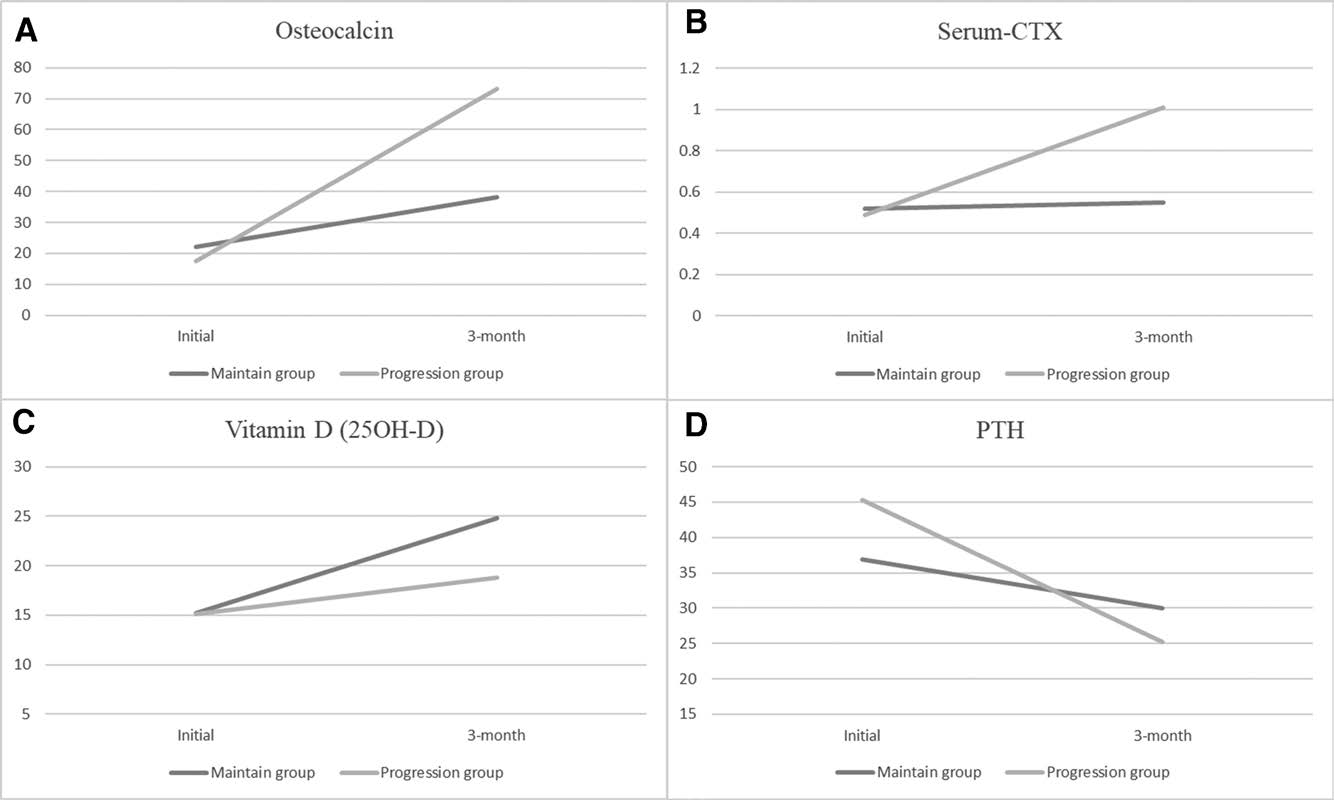
(A–D) Changes in the OC, serum CTX, vitamin D, and PTH between the maintain and progression groups recorded pretreatment and 3 mo after teriparatide treatment. CTX = C-terminal telopeptide of collagen, OC = osteocalcin, PTH = parathyroid hormone.

There were no significant differences in the BTM values between the 2 groups at the initial enrollment. The teriparatide treatment increased the total levels of OC (from 20.24 ± 16.53 to 50.50 ± 42.74, *P* = .001), serum CTX (from 0.51 ± 0.24 to 0.70 ± 0.54, *P* = .014), and vitamin D (from 15.16 ± 5.34 to 20.79 ± 7.96, *P* = .001); however, the PTH levels decreased (from 40.98 ± 21.71 to 28.96 ± 19.54, *P* = .001; Table 3). At the same time, in the “progression” group, the OC and serum CTX levels were significantly higher (*P* = .028 and .008, respectively) than in the maintain group. Interestingly, the vitamin D levels were significantly lower (*P* = .038) in the progression group than in the maintain group, although the difference between the pretreatment and the follow-up values was not significant (*P* = .077). The changes in the PTH levels before and after the treatment were not significant between the groups (*P* = .183 and *P* = .418, respectively); however, the “progression” group showed a significant decrease in the PTH values after the follow-up compared to the “maintain” group (*P* = .006; Table 3, Fig. 2).

**Table 3:** Summary of BTM values.

BTM (reference value)	Total	Maintain group	Progression group	*P* value*
Osteocalcin, ng/mL (11–48)				
Pretreatment	20.24 ± 16.53	22.00 ± 19.30	17.64 ± 9.38	.362†
F/U	50.50 ± 42.74	38.18 ± 21.93	73.09 ± 60.27	.028†
ΔF/U–pretreatment		16.18 ± 31.11	56.07 ± 53.46	.001†
*P* value*	.001‡			
Serum CTX, ng/mL (0.01–1)				
Pretreatment	0.51 ± 0.24	0.52 ± 0.20	0.49 ± 0.29	.616†
F/U	0.70 ± 0.54	0.53 ± 0.33	1.01 ± 0.69	.008†
ΔF/U–pretreatment		0.01 ± 0.41	0.53 ± 0.62	.001†
*P* value*	.014‡			
Vitamin D (25OH-D), ng/mL				
Pretreatment	15.16 ± 5.34	15.22 ± 5.87	15.09 ± 4.68	.940†
F/U	20.79 ± 7.96	24.79 ± 10.03	18.78 ± 9.37	.038†
ΔF/U–pretreatment		9.41 ± 14.27	3.69 ± 9.18	.077†
*P* value*	.001‡			
PTH, pg/mL				
Pretreatment	40.98 ± 21.71	36.83 ± 23.47	45.26 ± 16.86	.183†
F/U	28.96 ± 19.54	30.00 ± 22.24	25.22 ± 14.32	.418†
ΔF/U–pretreatment		–4.25 ± 24.09	–23.29 ± 16.45	.006†
*P* value*	.001‡			

Within each group, changes in the total calcium, ionized calcium, and Mg levels were different before and after the treatment; however, only the ionized calcium and Mg levels were significantly different between the 2 groups: in the “progression” group, the ionized calcium levels increased (*P* = .001), while the Mg levels decreased after the teriparatide treatment (*P* = .002; Table 4).

**Table 4:** Summary of blood test results.

Laboratory (reference value)	Maintain group	Progression group	*P* value*
Total protein. g/dL (6–8.3)			
Pretreatment	6.59 ± 0.63	6.57 ± 0.52	.902
F/U	6.84 ± 0.58	6.80 ± 0.65	.911
ΔF/U–pretreatment	0.24 ± 0.79	0.30 ± 1.13	.502
Albumin, g/dL (3.5–5.2)			
Pretreatment	4.07 ± 0.34	3.62 ± 0.45	.002
F/U	3.99 ± 0.26	3.90 ± 0.26	.552
ΔF/U–pretreatment	-0.03 ± 0.41	0.00 ± 0.25	.065
BUN, mg/dL (8–23)			
Pretreatment	16.42 ± 3.99	22.12 ± 11.66	.088
F/U	15.49 ± 5.56	17.00 ± 6.01	.638
ΔF/U–pretreatment	–3.33 ± 8.01	–3.35 ± 5.02	.396
Creatinine, mg/dL (0.5–1.3)			
Pretreatment	0.70 ± 0.13	0.93 ± 0.67	.215
F/U	0.76 ± 0.34	0.86 ± 0.41	.610
ΔF/U–pretreatment	0.02 ± 0.15	–0.02 ± 0.25	.395
Blood glucose, mg/dL (60–100)			
Pretreatment	114.92 ± 29.88	122.18 ± 25.22	.537
F/U	95.90 ± 27.71	94.78 ± 62.06	.962
ΔF/U–pretreatment	–8.73 ± 48.87	–4.00 ± 13.45	.207
Inorganic phosphorus, mg/dL (2.5–5.5)			
Pretreatment	3.56 ± 0.58	3.31 ± 0.83	.212
F/U	3.67 ± 0.47	3.67 ± 0.70	.988
ΔF/U–pretreatment	0.31 ± 0.97	0.37 ± 0.74	.757
Total Ca, mg/dL (8.4–10.2)			
Pretreatment	9.20 ± 0.56	8.89 ± 0.49	.063
F/U	9.38 ± 0.57	9.54 ± 0.38	.285
ΔF/U–pretreatment	0.75 ± 2.11	0.66 ± 0.61	.852
Ionized-Ca, mEq/L (2.2–2.6)			
Pretreatment	2.35 ± 0.08	2.31 ± 0.14	.143
F/U	2.36 ± 0.16	2.51 ± 0.11	.001
ΔF/U–pretreatment	0.25 ± 0.72	0.2 0 ± 0.14	.751
Mg, mg/dL (1.9–2.5)			
Pretreatment	2.15 ± 0.33	2.25 ± 0.14	.186
F/U	2.10 ± 0.17	1.93 ± 0.16	.002
ΔF/U–pretreatment	0.25 ± 0.83	–0.20 ± 0.63	.061

A multivariate analysis was performed to determine the correlation between the progression of compression fracture and the pretreatment BTM values. This analysis showed that pretreatment OC, serum CTX, vitamin D, and PTH could not be used as factors to predict the progression of compression fractures.

### 3.4. Case illustration

**Figure 3.**
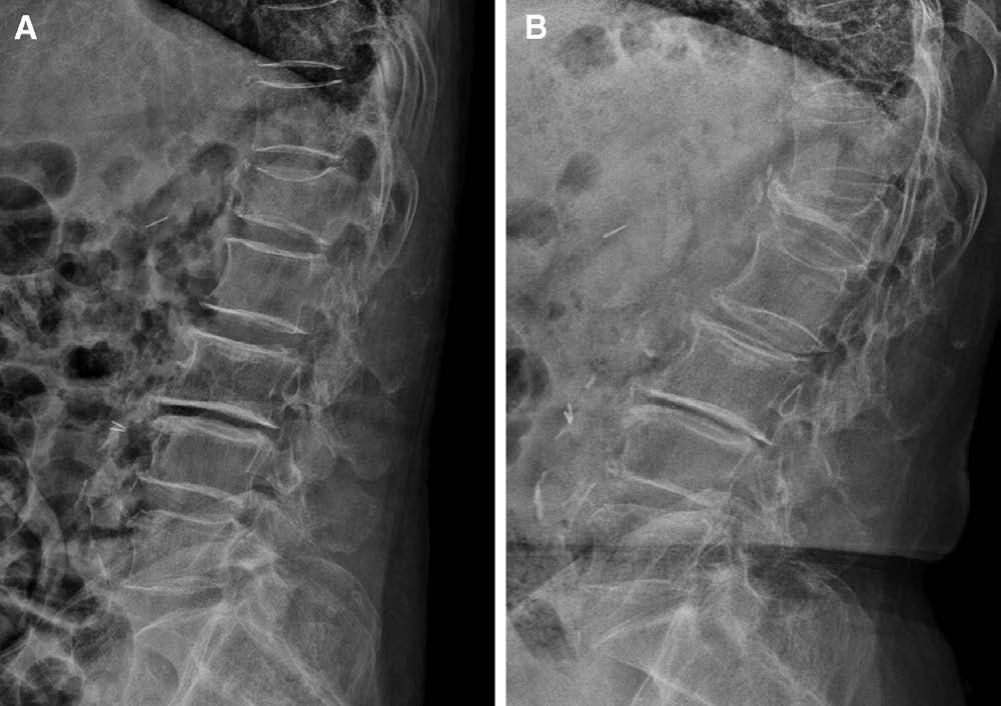
The case study of a 77-yr-old female patient with an L1 compression fracture treated with teriparatide. (A) The pretreatment lateral radiograph showing a VCR of 7.34% and a CA of 21.5º. The pretreatment BTM values were as follows: OC (4.38 ng/mL), serum CTX (0.21 ng/mL), vitamin D (11.23 ng/mL), and PTH (67 pg/mL). (B) The posttreatment lateral radiograph showing a VCR and CA progression of 65.15% and 24º, respectively, after 3.5 mo. The posttreatment BTM values were as follows: OC (29.18 ng/mL), serum CTX (0.42 ng/mL), vitamin D (24.19 ng/mL), and PTH (34 pg/mL). BTM = bone turnover markers, CA = Cobb angle, CTX = C-terminal telopeptide of collagen, OC = osteocalcin, PTH = parathyroid hormone, VCR = vertebral compression ratio.

A 77-year-old woman was admitted to our hospital with severe back pain. On physical examination, both lower extremities revealed normal strength. Radiological findings of the lumbar spine showed an L1 burst fracture. The CA and VCR were measured as 21.5º and 7.34%, respectively (Fig. 3A). The pretreatment BMD T score was–2.7 and the VAS score was 6. The pretreatment OC, serum CTX, vitamin D, and PTH levels were 4.38 ng/mL, 0.21 ng/mL, 11.23 ng/mL, and 67 pg/mL, respectively. After 3.5 months of teriparatide treatment, the VAS score improved to 3, but the compression fracture still progressed. The posttreatment CA and VCR values were 24º and 65.15%, respectively (Fig. 3B). The posttreatment OC, serum CTX, vitamin D, and PTH levels were 29.18 ng/mL, 0.42 ng/mL, 24.19 ng/mL, and 34 pg/mL, respectively. Consistent with our previous observations, the OC, serum CTX, and vitamin D levels increased, while the PTH levels decreased.

## 4. Discussion

Short-term conservative treatment with teriparatide is considered to be the first-line treatment for OVCFs without neurological deficits.^[4,10]^ Teriparatide promotes bone formation and improves bone strength and quality via the direct stimulation of osteoblasts, preventing osteoblast apoptosis, and increasing osteoblast activity.^[2–5,10]^ Consequently, teriparatide can rapidly decrease back pain compared to a placebo and treatment with antiresorptive agents.^[4]^ In our study, back pain was relieved after 3 months of teriparatide treatment (*P* = .001) regardless of the radiological outcomes. However, the effectiveness of short-term teriparatide treatment in preventing the progression of fractures remains uncertain. Kang et al^[2]^ reported that after 3 months of teriparatide treatment did not result in protective effects with respect to the progression of fracture in patients with OVCFs. In our study, the fractures progressed in 37.3% of OVCF segments after 3 months of teriparatide treatment. However, the aim of our study was not to identify whether short-term teriparatide exerts protective effects on the progression of fracture in patients with OVCF; rather we aimed to investigate whether changes in BTM parameters after the teriparatide treatment correlate with the fracture progression and evaluate whether these changes can be used to predict the fracture progression.

BTMs are the indicators of bone cell activity; their discovery has contributed to a marked improvement in the management of osteoporosis. BTMs are generally divided into 3 categories: BFMs, BRMs, and osteoclast regulatory proteins.^[6]^ Several studies have reported that significant increases in the OC and serum CTX levels were observed after teriparatide treatment, and their levels varied depending on dynamic changes in bone metabolism, as well as various stages of fracture healing.^[11–13]^ These studies revealed that the OC and serum CTX levels reflect active bone formation and resorption, respectively, after teriparatide treatment; however, these results are not sufficient for clinical application. Hong et al^[14]^ reported that when patients with OVCF without fracture progression were divided into teriparatide treatment and control groups, the OC and serum CTX levels increased in both groups, but there was no significant difference. In our study, changes in OC and serum CTX levels in response to the teriparatide treatment were significantly larger in the progression group than those in the maintain group (*P* = .001). Considering that the BRMs increase during the first 4 weeks after the fracture and then the BFMs increase,^[15,16]^ the large increase in OC and serum CTX after 3 months of teriparatide treatment could mean that new fractures indicating fracture progression and the resorption phase of the bone remodeling cycle. In contrast, the endogenous PTH levels reduced during the teriparatide treatment, possibly in response to the bone remodeling process.^[17]^ In our study, there was no difference in the endogenous PTH levels at the follow-up stage between the groups (*P* = .418); however, the decrease in PTH levels after teriparatide treatment was significantly larger in the progression group than in the maintain group (*P* = .006). This result reflects that negative feedback for endogenous PTH is maintained even after 3 months of a compression fracture and that more bone remodeling reactions occur in the progression group. We propose that the further increases in the levels of OC and serum CTX, as well as further suppression of the endogenous PTH levels after 3 months of teriparatide treatment, reflect more bone remodeling reactions, including resorption and formation phase, which is a response to the progression of compression fractures.

PTH increases the conversion of 25-hydroxyvitamin D [25(OH)D] into 1,25 dihydroxyvitamin D [1,25(OH)_2_D]. Consequently, the vitamin D (25OH-D) concentration usually decreases after the teriparatide treatment.^[18,19]^ In our study, after the teriparatide treatment, the concentration of vitamin D (25OH-D) increased in both groups; however, this increase was smaller in the progression group than in the maintain group (*P* = .038). We believe that this was the combined effect of calcium and cholecalciferol supplementation, in addition to the teriparatide treatment during the study period, and the low vitamin D (25OH-D) concentration in the “progression” group could be the result of active bone healing.

Serum Mg is an important cofactor for enzymes involved in the normal synthesis of the bone matrix. Several studies have suggested that low serum Mg levels are associated with an increased risk of osteoporosis.^[20–22]^ In our study, the serum Mg levels were lower in the progression group than in the maintain group (*P* = .002; Table 4), although the Mg levels in both groups remained within the normal range. Given the evidence of the role of Mg in osteoporosis, Mg supplementation may increase bone density and delay bone loss.

High bone turnover is associated with the increased risk of osteoporotic fractures, and BTMs might be a useful tool to predict fractures independent of the BMD.^[6,23–25]^ Our results suggest that the pretreatment BTM levels cannot predict the progression of OVCFs. Furthermore, there is a large variability between individuals due to difference in age and physiological maturity and the multiple methodologies used for analysis, adding to the unpredictability of using BTMs as predictive markers for clinical applications; however, changes in BTM values during the teriparatide treatment could provide useful information for predicting the progression of OVCFs. If there is a significant increase in the OC and serum CTX levels, as well as a significant decrease in the PTH levels during the teriparatide treatment in patients with OVCF, clinicians should frequently perform clinical and radiological evaluations of patients and should consider other treatment options such as transitioning to denosumab,^[26]^ percutaneous vertebroplasty, and surgical fixation.

This retrospective study has several limitations. Frequent lab tests regarding osteoporosis are more expensive than checking a simple radiograph, and the clinician should obtain the patient’s consent regarding the cost of the lab tests. The study analyzed a small number of patients and collected only short-term follow-up results after the teriparatide treatment. Consequently, it is difficult to generalize about all patients with OVCFs from a small sample. We had no control group to compare radiological outcomes and BTM values in patients not treated for OVCF. Moreover, evaluation of the increase or decrease in BTM values is not quantitative or ideal for clinical decision-making when predicting fracture progression. Further randomized prospective and comparative studies with a longer follow-up period are needed to fully understand the progression of OVCFs.

## 5. Conclusion

In patients with OVCFs of the thoracic and lumbar spine, changes in the OC and serum CTX levels were significantly higher in the progression group than in the maintenance group. In addition, the serum PTH levels were significantly reduced in the progression group after 3 months of teriparatide treatment. Based on our results, we recommend a thorough evaluation of BTM values during teriparatide treatment in patients with OVCF, that is, if there is a significant increase in the OC and serum CTX levels, coinciding with a significant decrease in the PTH concentration during the teriparatide treatment, the clinician should assume the progression of fracture, conduct radiological evaluations, and consider other treatment options.

## Author contributions

Conceptualization: Bong Ju Moon.

Data curation: Gwang-Jun Lee.

Funding acquisition: Bong Ju Moon, Jung-Kil Lee.

Investigation: Moon-Soo Han, Seul-Kee Lee.

Software: Moon-Soo Han.

Supervision: Bong Ju Moon.

Writing – original draft: Moon-Soo Han.

## Footnotes

* A paired *t* test was used for analysis.

† A comparison of mean values between the maintain and progression groups.

‡ A comparison of mean values between the pretreatment and follow-up.

* A paired *t* test was used for analysis.

† A comparison of mean values between the maintain and progression groups.

‡ A comparison of mean values between the pretreatment and follow-up.

* A comparison of mean values between the maintain and progression groups
